# Impact of Non-Invasively Induced Motor Deficits on Tibial Cortical Properties in Mutant *Lurcher* Mice

**DOI:** 10.1371/journal.pone.0158877

**Published:** 2016-07-07

**Authors:** Alena Jindrová, Jan Tuma, Vladimír Sládek

**Affiliations:** 1 Department of Anthropology and Human Genetics, Faculty of Science, Charles University, Prague, Czech Republic; 2 Department of Pathophysiology, Faculty of Medicine in Pilsen, Charles University, Pilsen, Czech Republic; 3 Laboratory of Neurodegenerative Disorders, Biomedical Centre, Faculty of Medicine in Pilsen, Charles University, Pilsen, Czech Republic; Université de Lyon—Université Jean Monnet, FRANCE

## Abstract

It has been shown that *Lurcher* mutant mice have significantly altered motor abilities, regarding their motor coordination and muscular strength because of olivorecebellar degeneration. We assessed the response of the cross-sectional geometry and lacuno-canalicular network properties of the tibial mid-diaphyseal cortical bone to motor differences between *Lurcher* and wild-type (WT) male mice from the B6CBA strain. The first data set used in the cross-sectional geometry analysis consists of 16 mice of 4 months of age and 32 mice of 9 months of age. The second data set used in the lacunar-canalicular network analysis consists of 10 mice of 4 months of age. We compared two cross-sectional geometry and four lacunar-canalicular properties by I-region using the maximum and minimum second moment of area and anatomical orientation as well as H-regions using histological differences within a cross section. We identified inconsistent differences in the studied cross-sectional geometry properties between *Lurcher* and WT mice. The biggest significant difference between *Lurcher* and WT mice is found in the number of canaliculi, whereas in the other studied properties are only limited. *Lurcher* mice exhibit an increased number of canaliculi (*p* < 0.01) in all studied regions compared with the WT controls. The number of canaliculi is also negatively correlated with the distance from the centroid in the *Lurcher* and positively correlated in the WT mice. When the *Lurcher* and WT sample is pooled, the number of canaliculi and lacunar volume is increased in the posterior I_max_ region, and in addition, midcortical H-region exhibit lower number of canaliculi, lacuna to lacuna distance and increased lacunar volume. Our results indicate, that the importance of precise sample selection within cross sections in future studies is highlighted because of the histological heterogeneity of lacunar-canalicular network properties within the I-region and H-region in the mouse cortical bone.

## Introduction

Bone adapts to its mechanical loading history during life and therefore reflects differences in habitual loading [1–7]. The relationship between mechanical loading and bone morphology has been supported at several hierarchical designs, which range from analyses of the structural properties of long bones, including the appendicular skeleton configuration [8, 9], macroscopic morphology of whole bones [10] and cross-sectional geometry (CSG) properties [6, 11–16], to analyses of the microstructural properties of long bones, such as the trabecular orientation [17] and spatial distribution of osteocytes and the lacunar-canalicular network (LCN) in cortical bone tissue [18–23]. The most important response of bone tissue to mechanical stimuli in long bone diaphysis was demonstrated in CSG properties [24, 25], as well as the spatial distribution of the LCN [26]. In addition to this general observation, it remains unclear how the cortical bone distribution in long bone diaphysis and its microstructural properties are related to each other [27]. Therefore, we assessed the response of structural (CSG) and microstructural (LCN) properties to mechanical loading differences in two different habitual loading models of mice.

Previous studies have demonstrated the effect of mechanical loading and unloading on bone morphology mainly using non-physiological loading models or applied strains that were outside the normal expected range of loading experienced by healthy animals. These experimental studies have included, for example, surgical interventions [28], the application of artificial mechanical overload *in vivo* [29] and mechanical unloading [22]. In contrast, as suggested for example by Pearson and Lieberman [30], it is not completely understood how the CSG and LCN respond to different mechanical loadings induced in the optimum physiological state and expected physiological magnitude. Therefore, we used mice with olivocerebellar motor disorder (*Lurcher*, Lc) and their healthy controls to assess the effects of differences in mechanical loading on bone cortical tissue without the impact of an invasive intervention.

*Lurcher* (Lc) mice (gene symbol: *Grid2*^*Lc*^) constitute a natural model of hereditary olivocerebellar degeneration [31, 32]. The semi-dominant heterozygous *Lurchers* (+/Lc) suffer from progressive loss of cerebellar Purkinje cells, followed by the death of granule cells and inferior olive neurons as a result of the disappearance of connective pathways [33]. The degenerative process is initiated at postnatal day 8 (P8) and finalized at P90 when loss of Purkinje cells is virtually complete and only 10% of granule cells and 30% of inferior olive neurons remain [34]. Due to olivocerebellar degeneration, *Lurcher* mice suffer from cerebellar ataxia and related motor changes [35–42], such as short steps, vertical displacement of the hip and an inability to produce continuous step cycles without stumbling [31, 39, 40]. The most prominent disturbances in Lc mutants are a dynamic tremor and a frequent loss of equilibrium that is compensated, in part, by a spayed stance to preserve balance [39]. Moreover, *Lurcher* mice often initiate forelimb stepping movements before the center of gravity is transferred forward by the hindlimbs [39]. The altered motor performance also affects the muscle fragility, which is significantly increased in Lc mice [43]. In addition, *Lurchers* have irregular electromyography (EMG) pattern during walking [39]. Lc mutants are commonly used to investigate the impact of olivocerebellar degeneration on motor performance [36, 37, 39, 41, 42] and motor learning [36, 38, 42]; however, it would also be interesting to determine how these physiologically induced motor changes affect CSG and LCN properties. In general, we suggest that the altered motor performance would be reflected in both the CSG and LCN properties; however, this hypothesis has not been tested to date. In this study, we determine whether the changes in mechanical loading induced by the non-invasively altered motor performance in Lc mice affect the CSG and LCN properties of cortical tissue in long bone diaphysis.

It has been demonstrated that long bone diaphysis behaves similar to engineering beams [44] in which the geometric structure of their cross sections is sensitive to the amount and pattern of a particular mechanical loading [45]. In this study, we estimated the compressive and tensile mechanical loading using a cortical area (CA) in cross section and the bone resistance to torsional and bending loading using a polar section modulus (Zp) to assess the differences in the CSG properties between Lc mutants and WT controls.

It is widely accepted that osteocytes are the most mechanosensitive cells in bone that are involved in the transduction of a mechanical signal into a biological response [46, 47]. There is great interest in how the spatial properties of osteocytes and its processes, as reflected by their lacunae and canaliculi within cortical bone tissue, may be related to the pattern of mechanical loading. It has been demonstrated that the lacunar shape and volume are variable within a transversal cross section [22, 23, 27] which might be caused by general mechanical loading differences [27]. For example, it has been demonstrated, that lacunae were more elongated and preferentially oriented parallel to the principle mechanical loading direction under uni-directional loading compared with more spherical and not axially oriented lacunae in a multi-direction loading environment [19]. However, the association between the pattern of mechanical loading and LCN formation remains unclear. Therefore, we analyzed the spatial distribution of the LCN in Lc mice and determined whether the altered mechanical loading induced by the olivocerebellar motor deficit of mutants also affects other LCN parameters.

It has been reported that the cortical bone microstructure within a tibial and femoral cross section is not homogenous [22, 23]. It is likely that the strains increase in magnitude as one moves further from the neutral bending axis; thus, the cells should be accommodated to different strains at each point across the long bone section [48]. This finding also corresponds with the pattern of localized variation identified in the previous study, in which the midcortical region of tibial diaphysis cross sections exhibited a disorganized “woven-like” appearance with visibly more lacunae [22]. Here, we analyze the differences in the LCN properties according to the distance from the neutral axis and within the periosteal, midcortical and endosteal localization, as well as with respect to maximum and minimum second moment of area (I_max_, I_min_).

Various high resolution imaging technologies have been applied to investigate the spatial properties of the LCN of long bone tissue, including confocal laser scanning (CLS) [49–51], synchrotron radiation (SR) based micro-CT [22, 52] and nano-CT [19, 21]. Considering the pattern of regional variation in the spatial parameters of the LCN, we concentrated on confocal laser scanning to combine the advantages of a high resolution imaging technology with the ability to analyze the LCN within the entire cross section, as well as the potential to estimate the CSG properties of the studied sections via high resolution images.

## Materials and Methods

### Data set

The first data set used in the analyses of the CSG properties consisted of 16 male mice (Lc = 7; WT = 9) at 4 months (4M) of age and 32 male mice (Lc = 16; WT = 16) at 9 months (9M) of age from the B6CBA strain. The second data set used in the LCN analysis consisted of 10 male mice (Lc = 5; WT = 5) at 4 months of age previously studied in the 4M CSG analyses. We analyzed 1160 single lacunae (Lc = 572; WT = 588) randomly selected from the individuals from the second data set.

Mice used in this study were obtained by crossing WT females with Lc males. All animals were maintained in plastic cages (20 × 25 × 14 cm), 2–4 animals per cage in the room with controlled temperature (22–23°C) and humidity (50–60%) with a 12/12 h light/dark cycle. Food (standard commercial pellet diet) and water were available *ad libitum*. The experiments were performed in compliance with the EU Guidelines for scientific experimentation on animals and with the permission of the Ethical Commission of the Faculty of Medicine in Pilsen, Charles University, Czech Republic.

### Motor performance

All tests of motor performance were conducted at the Department of Pathophysiology of Charles University, Medical Faculty in Pilsen, Czech Republic. The motor performance of the experimental animals was examined using the continuously accelerating rotarod (motor coordination) and the horizontal wire (strength performance) as previously described [53]. For the rotarod test, the mouse was placed on the rotarod cylinder (RotaRod Advanced, TSE Systems Ltd., Bad Homburg, Germany) with its head in the opposite direction of the rotation. The fall latencies were measured with a maximal latency of 120 seconds. The horizontal wire test enables the assessment of muscular strength and neuromuscular functions. The animal was suspended by its two forepaws in the middle of the horizontal wire (1 mm diameter, 43 cm length) 40 cm above a desk covered with a soft pillow. The fall latencies were measured. If the animal did not fall down within 60 seconds, the trial was terminated. The rotarod and horizontal wire tests were repeated four times at 8 minute intervals for each mouse. The mean latencies of the 4 trials were calculated.

As indicated by the motor coordination and strength performance, the studied Lc mutants exhibited an approximately 86% decrease in motor performance. The decrease in motor performance between the Lc and WT mice was also identified in our total CSG data set in which the Lc mutants exhibited a 29.9% reduction in motor coordination and an 83% reduction in strength performance compared with the WT controls. Thus, our results regarding the decreased motor performance in Lc mutants are in agreement with previous observations, which also demonstrated significant motor dysfunction in Lc cerebellar mutants compared with healthy WT mice [35–42].

### Preparation of bone specimens and fluorescence staining

The animals were sacrificed by cervical dislocation, and the left tibia was dissected. Cortical bone sections were prepared and stained using a protocol for undecalcified bone [54]. Briefly, immediately after dissection, the bones were fixed in Karnovsky’s fixative and cut with a diamond blade saw (Buehler, Co., Dusseldorf, Germany) in the transverse plane at 50% of the bone biomechanical length. The cortical sections were ground to a final thickness of ∼ 50 μm using Carbimet paper discs (800 and 1200 grit; Buehler, Co., Dusseldorf, Germany), dehydrated in ascending graded ethanol and stained with fluorescein isothiocyanate (FITC; Sigma-Aldrich, Co., St. Louis, MO, USA).

### Confocal laser scanning images

The stained sections were imaged using a Leica TCS SP2 confocal microscope with an Acousto-Optical Beam Splitter system (AOBS). The scanning parameters for the CSG analysis were as follows: 10× lens, 0.4 numerical aperture, Argon-Argon/Krypton laser wavelength excitation of 488 nm, 500–560 nm emission, pinhole set at 1 Airy unit, laser intensity set at the minimum power level and 20% on the Acusto-Optical Tunable Filter (AOTF), 1024 × 1024 resolution, which provide a field of view of 1500 × 1500 μm (1 pixel = 1.465 μm), and 1.78 μm step size. The high resolution scanning for the LCN analysis differed in the following parameters: 63× lens, 1.40 numerical aperture, 1 pixel = 0.116 μm and 0.25 μm step size for a scan depth of 50 μm. After scanning the stacks were deconvoluted using SVI Huygens software (Scientific Volume Imaging; The Netherlands).

### CSG properties

The cross section images were analyzed using NIH ImageJ (version 1.47d) [55], which contains a MomentMacro [56] modified for use with histological sections (https://www.natur.cuni.cz/biologie/servisni-laboratore/laborator-konfokalni-a-fluorescencni-mikroskopie/imagej-macros/moment-macro-j/view). Both, the periosteal and endosteal contours were manually traced [57], and the bone structural parameters were subsequently calculated: cross-sectional cortical area (CA), second moment of area (I) and polar section modulus (Zp). The cortical area is a measure of the amount of cortical bone in a cross section and determines the rigidity and strength of the long bone under pure axial (compressional or tensile) loading. The second moment of area can be calculated around any axis through a section; however, it is most commonly measured around the anatomical axes of the bone (medio-lateral, antero-posterior) or as the maximum and minimum second moment of area (I_max_, I_min_) [58]. Here we used the axes of the maximum and minimum second moment of area calculated by the software program to divide the cross section into smaller regions related to particular axes. The polar section modulus determines the cross-sectional torsional strength (twice average bending strength) [45]. Polar section modulus can also estimate the overall bone robustness when appropriately adjusted for body size. The applied force for both the axial and bending loading is proportional to the body mass; specifically, the force exerted by the body mass and bending loading is also proportional to the bone length determinates section location [59]. Therefore, we scaled the cortical area (CA) with the body mass and the polar section modulus (Zp) with the product of the body mass and bone length for each individual according to a standard protocol [59]. The body mass was estimated using a mean value calculated from the three particular body mass estimates obtained from each individual on three consecutive days prior to sacrifice. The biomechanical length was measured parallel to the longitudinal axis of each tibia between the proximal and distal articular surfaces using a digital caliper.

### Cross-sectional regions

**Fig 1:**
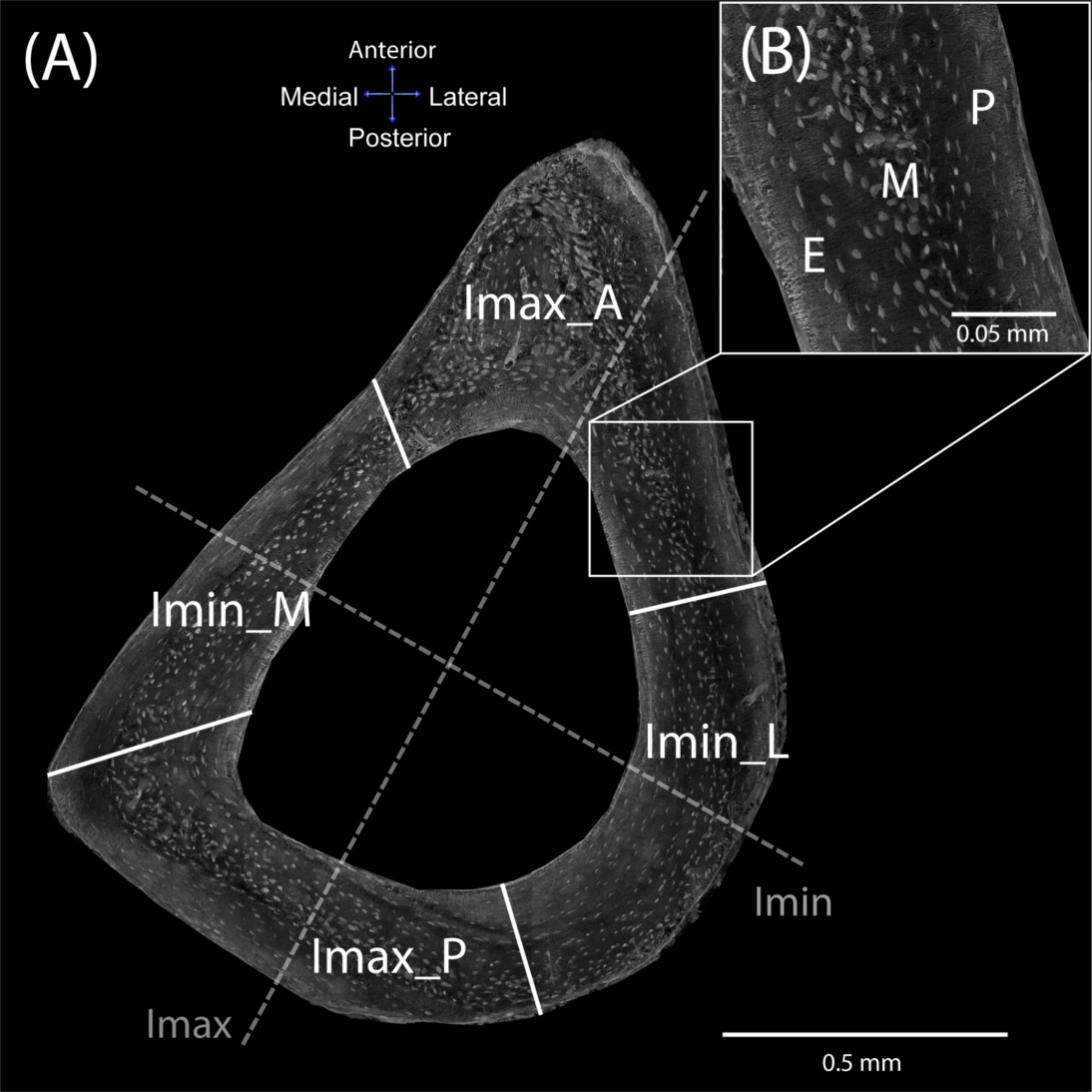
Location of selected regions within the mouse tibial mid-diaphyseal cross section. (A) I-regions (I_max__A–anterior region around maximum second moment of area, I_max__P–posterior region around the maximum second moment of area, I_min__M–medial region around the minimum second moment of area, and I_min__L–lateral region around the minimum second moment of area) and (B) H-regions (P–periosteal, M–midcortical, E–endosteal; the section shows histological details for the periosteal, midcortical and endosteal regions selected within each I-region).

Fig 1 shows the division of each cross section according to the I-regions and H-regions to further localize each studied lacunae in the analysis. The I-regions were defined using the maximum and minimum second moment of area and anatomical axes. Each cross section was divided into four I-regions: I_max__A (anterior region around the maximum second moment of area), I_max__P (posterior region around the maximum second moment of area), I_min__M (medial region around the minimum second moment of area), and I_min__L (lateral region around the minimum second moment of area). The boundary between each I-region was created using the radial line plotted in the half angle between the two adjacent I axes.

As discussed in the Introduction and shown in Fig 1B, lacunae are not histologically homogenous in a mouse cross section; however, three regions can be distinguished according to the periosteal and endosteal localization. Thus, three H-regions were defined in each cross section: endosteal (E), midcortical (M; woven-like histological structure encased between the endosteal and periosteal regions), and periosteal (P) regions. The boundary between the midcortical and periosteal/endosteal regions was created using manual tracking of the disorganized woven-like structure within the cross section. The boundary line was conducted along the first visible disorganized lacunae of the midcortical region.

### Lacunar-canalicular network properties

The distance of each lacuna from the cross-sectional centroid, endosteum and periosteum was measured using a measuring tool in NIH ImageJ (version 1.47d) [55]. The distance from the centroid was measured between the centroid and lacuna center. The distance of the lacuna from the external endosteal and periosteal contours was measured using an axis that ran radially from the centroid through the center of the lacuna to the periosteum.

**Fig 2:**
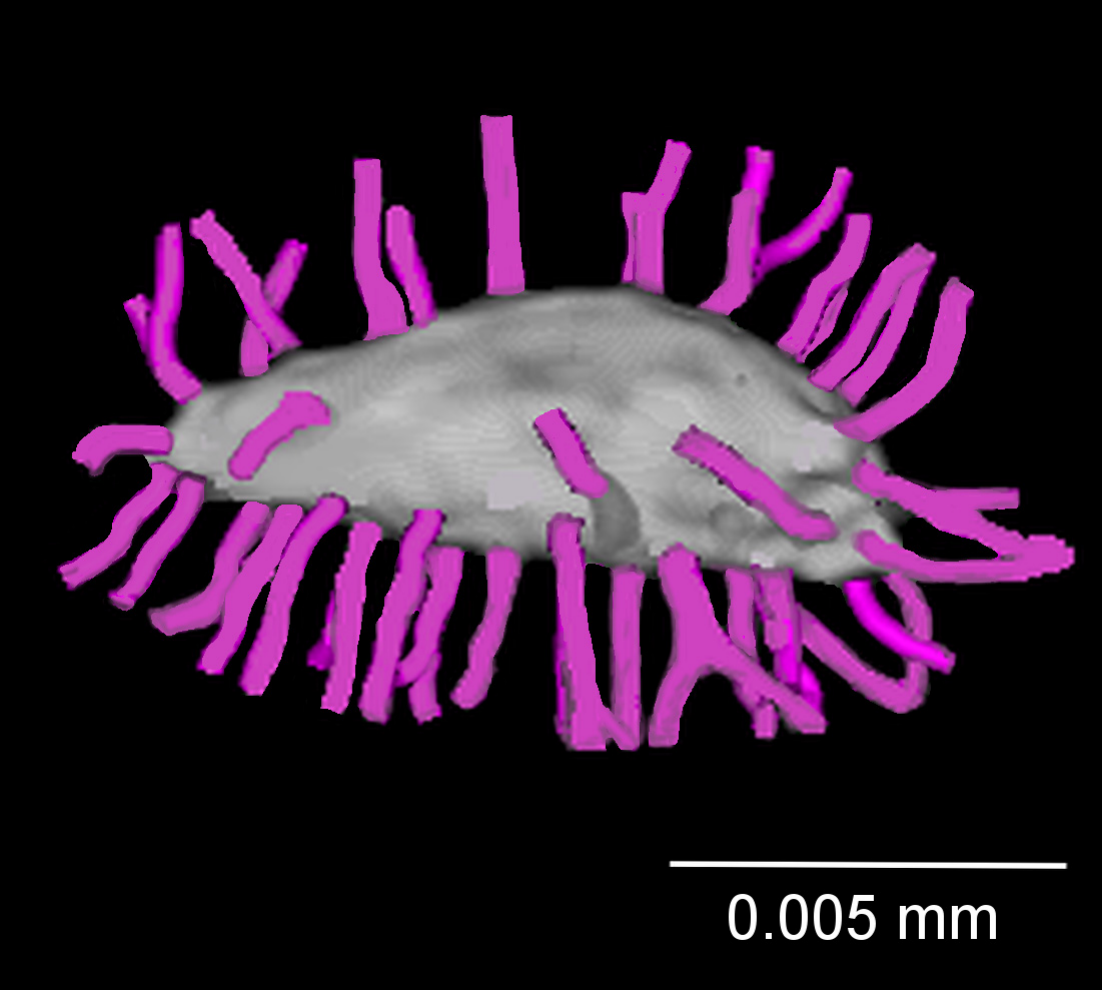
The 3D-reconstructed model of lacuna and its canaliculi manually traced (violet lines) in the number of canaliculi analysis.

The number of canaliculi that radiated from each lacuna (N.Ca) was counted using Fiji (version 1.48v) [60] and plugin Simple neurit tracer [61]. The lacuna was analyzed by rotating the 3D-reconstructed model (Fig 2) created by the software program. The visualization of the number of canaliculi distributed within the cross section was performed using Nemetschek Allplan (Munich, Germany).

The lacunar volume (Lac.V) was analyzed using NIH ImageJ (version 1.47d) [55] and the plugin Volumest [62]. The volume was calculated by manual tracing of the lacunar external contours on each image of the stack. The coefficient of error calculated by the software was between 0.015 and 0.04 (data not shown).

The orientation of each lacuna was measured as the absolute value of the angular deviation (−90° to 90°) of the first lacunar length axis (longest) from the periosteal external contour. The angular values were measured according to axis that ran from the centroid through the center of the lacuna to the periosteum. An orientation value of 0° represents a lacuna oriented parallel with the periosteal contour, and orientation values that approach 90° are increasingly perpendicular in orientation to the periosteum.

The lacuna to lacuna distance (Lac.Dist) was measured using a measuring tool in NIH ImageJ (version 1.47d) [55] in each cross-sectional area by counting the distance between the centers of the neighboring lacunae in a small 3D block that contained approximately 10 lacunae.

### Statistical analysis

All statistical analyses were performed using Statistica 12 for Windows (StatSoft, Inc.). The CSG measurements were analyzed using ANOVA with a post-hoc Fisher’s LSD adjustment for the two factors: treatment (Lc and WT) and age (4M and 9M). All CSG data are presented as the mean and standard deviation (SD). We also used ANOVA with a post-hoc Fisher’s LSD adjustment to analyze the LCN measurements for the three factors: treatment (Lc and WT), I-region (I_max__A, I_max__P, I_min__L, I_min__M) and H-region (endosteal, midcortical, periosteal). We revealed that our raw data meet the condition of homogeneity of variances but meet only partially the condition of normality. Hence, we conducted several additional tests such as *p*-value correction (see details in Skedros et al. [27]), the ANOVA with logarithmic transformation of the raw data, and non-parametric tests (Kruskal-Wallis) that showed consistent pattern of significances with our ANOVA results based on the raw data. Therefore, we assumed that the effect of normality does not affect our main conclusion and the results based on the raw data can be used as they may also serve as a reference for future studies. To examine the relationship between the LCN parameters and the distance from the centroid, a linear least-square regression analysis was performed. The differences were considered statistically significant if *p* < 0.05.

## Results

### Body size and CSG analysis

The differences in the body mass and biomechanical length between the Lc mutants and WT controls, as well as the 4M and 9M age groups are shown in Table 1. The body mass significantly increases with age in both the Lc (*p* < 0.001) and WT (*p* = 0.003) mice. The Lc mutants also exhibit significantly lower BM compared with the WT controls in both age groups (*p*_*(4M)*_ < 0.001; *p*_*(9M)*_ < 0.001). The biomechanical length is not significantly different with age in the Lc mice (*p* = 0.718) or WT controls (*p* = 0.249); however, the Lc mice have significantly shorter tibiae compared with the WT controls in both age comparisons (*p*_*(4M)*_ = 0.019; *p*_*(9M)*_ = 0.014). Thus, given the pattern of significant findings, size differences may play a role in the interpretation of the CSG results; therefore, all studied CSGs have to be also appropriately scaled to body size.

**Table 1:** Descriptive statistics for the body and CSG properties between the Lc mutants and WT controls from the first data set.

Parameter	B6CBA 4 months			B6CBA 9 months				
	*Lurcher*	Wild type		*Lurcher*	Wild type		*p*-value	*p*-value
	n = 7	n = 9		n = 16	n = 16		4 vs. 9	4 vs. 9
	Mean ± SD	Mean ± SD	*p*-value	Mean ± SD	Mean ± SD	*p*-value	months Lc	months WT
BM	26.18 ± 1.38	29.62 ± 1.67	< 0.001***	29.41 ± 2.23	32.00 ± 1.56	< 0.001***	< 0.001***	0.003**
BML	17.42 ± 0.41	17.85 ± 0.32	0.019*	17.36 ± 0.34	17.68 ± 0.36	0.014*	0.718	0.249
CA	70.30 ± 1.65	75.12 ± 6.55	0.101	76.37 ± 7.10	78.57 ± 4.59	0.281	0.023*	0.154
Zp	21.70 ± 2.98	24.69 ± 2.41	0.102	25.37 ± 5.03	27.45 ± 2.40	0.108	0.029*	0.073
CAstd	26.92 ± 1.51	25.42 ± 2.52	0.126	26.00 ± 1.87	24.59 ± 1.68	0.042*	0.294	0.302
Zpstd	4.75 ± 0.5	4.67 ± 0.4	0.779	4.94 ± 0.7	4.86 ± 0.4	0.680	0.474	0.443

BM–body mass (g), BML–biomechanical length (mm), CA–cortical area (mm2 × 102), Zp–polar section modulus (mm3 × 102), CAstd–standardized CA (mm2/g × 103), Zpstd–standardized Zp (mm3/g × mm × 104)

* *p* < 0.05

** *p* < 0.01

*** *p* < 0.001.

The differences in the CSG properties between the Lc mice and WT controls, as well as the age groups are shown in Table 1. In general, the differences in the CSG properties between the Lc mice and WT controls are less significant and consistent than the body size parameters. For the effect of age; the cortical area (*p* = 0.023) and the section modulus (*p* = 0.029) in the Lc mice exhibit significant increases between the 4M and 9M age groups; however, the significances are lost when an appropriate size standardization is applied.

### Number of canaliculi

**Fig 3:**
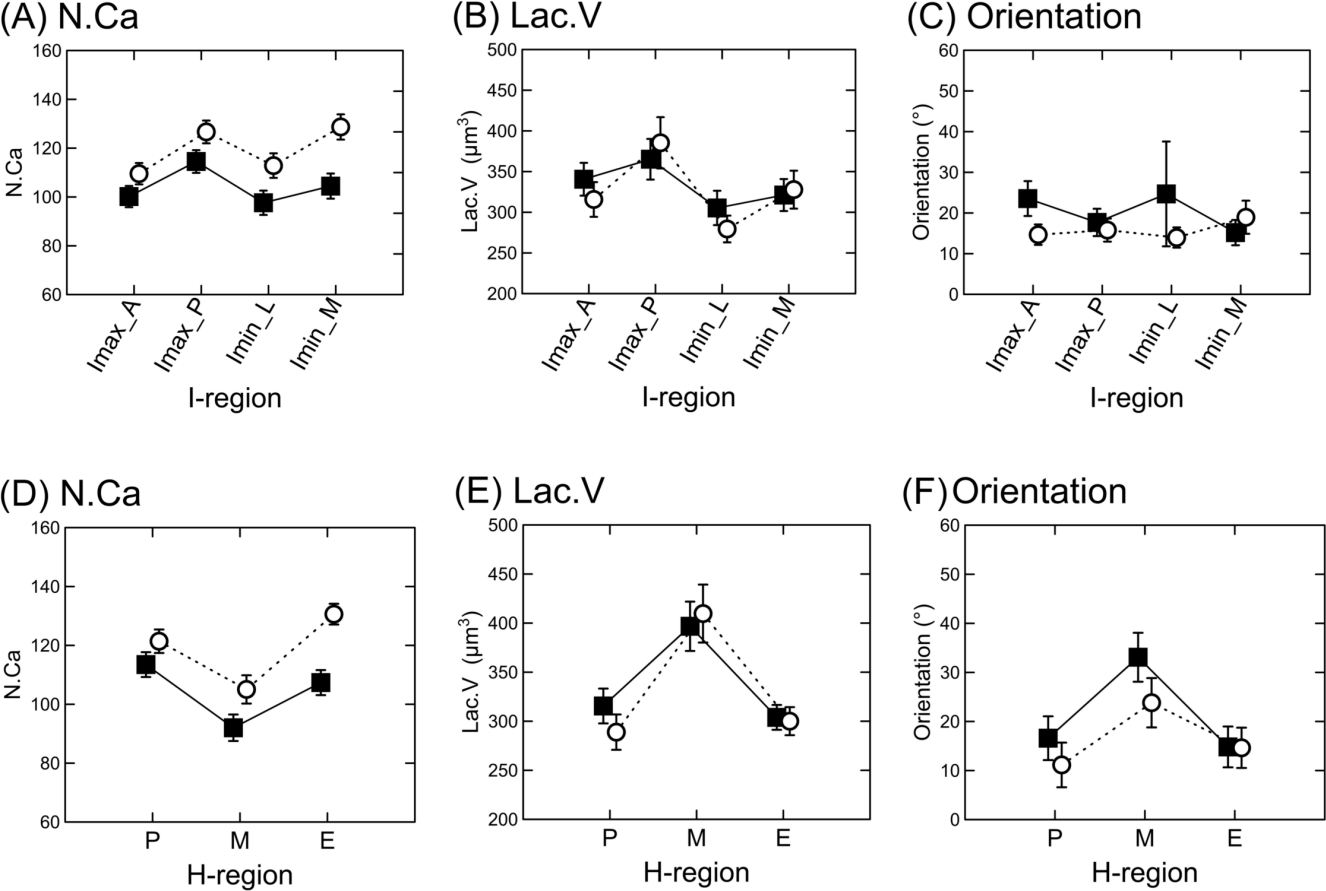
The mean of N.Ca (A, D), Lac.V (B, E) and lacunar orientation (C, F) by the I-regions and H-regions in the Lc mutants and WT controls from the second data set (Lc: open circles; WT: closed squares; vertical bars denote the 0.95 confidence intervals of the means; see abbreviations in Table 2).

Table 2 summarizes the differences in the number of canaliculi (N.Ca) between the Lc and WT samples by the I-regions and H-regions. N.Ca is significantly increased (*p* < 0.001) in the Lc mutant mice compared with the healthy WT controls when averaged over all regions (i.e., in pooled regions) as well as in all studied I-regions (Fig 3A). The Fisher’s LSD post-hoc tests indicate that N.Ca within both Lc and WT mice is significantly lower in region I_max__A and I_min__L and significantly increased in the region I_max__P and I_min__M when compared with the other I-regions. Similarly, N.Ca is significantly increased in the Lc mutant mice in all H-regions compared with the WT controls (Fig 3D). The Fisher’s LSD post-hoc tests indicate that N.Ca within the Lc mutants is significantly increased in the endosteal region and significantly lower in the midcortical region. The periostal regions within the WT controls have significantly increased N.Ca, whereas the midcortical regions have significantly lower N.Ca.

**Table 2:** Descriptive statistics for the number of canaliculi (N.Ca), lacunar volume (Lac.V) and orientation between the Lc mutants (n = 5) and WT controls (n = 5) from the second data set (letters in superscript indicate the Fisher’s LSD post-hoc significances between the I- and H-regions at *p* < 0.05).

Region	Average N.Ca			Average Lac.V (μm^3^)			Average orientation (°)		
	Lc	WT	*p*-value	Lc	WT	*p*-value	Lc	WT	*p*-value
All regions	120.54	104.99	< 0.001***	326.93	334.69	0.313	15.68	20.41	0.010*
I_max__A	110.12^b^^,^^c^^,^^d^	98.63^b^^,^^d^	< 0.001***	315.72^b^^,^^c^	340.59^c^	0.083	14.67	23.54^d^	0.010*
I_max__P	126.74^a^^,^^c^	116.59^a^^,^^c^^,^^d^	0.002**	385.36^a^^,^^c^^,^^d^	365.15^c^^,^^d^	0.183	15.79	17.68	0.602
I_min__L	118.35^a^^,^^b^^,^^d^	98.42^b^^,^^d^	< 0.001***	279.48^a^^,^^b^^,^^d^	305.31^a^^,^^b^	0.102	13.96	24.68^d^	0.004**
I_min__M	129.79^a^^,^^c^	106.35^a^^,^^b^^,^^c^	< 0.001***	327.80^b^^,^^c^	321.16^b^	0.688	18.95	15.17^a^^,^^c^	0.339
Periosteal	121.40^f^^,^^g^	113.48^f^^,^^g^	0.007**	288.86^f^	315.53^f^	0.047*	11.14^f^	16.85^f^	0.075
Midcortical	105.04^e^^,^^g^	92.02^e^^,^^g^	< 0.001***	409.68^e^^,^^g^	396.75^e^^,^^g^	0.371	22.97^e^^,^^g^	31.77^e^^,^^g^	0.011*
Endosteal	130.60^e^^,^^f^	107.36^e^^,^^f^	< 0.001***	300.07^f^	303.92^f^	0.755	14.27^f^	14.84^f^	0.847

^a^ Imax_A

^b^ Imax_P

^c^ Imin_L

^d^ Imin_M

^e^ periosteal

^f^ midcortical

^g^ endosteal

* *p* < 0.05

** *p* < 0.01

*** *p* < 0.001.

**Fig 4:**
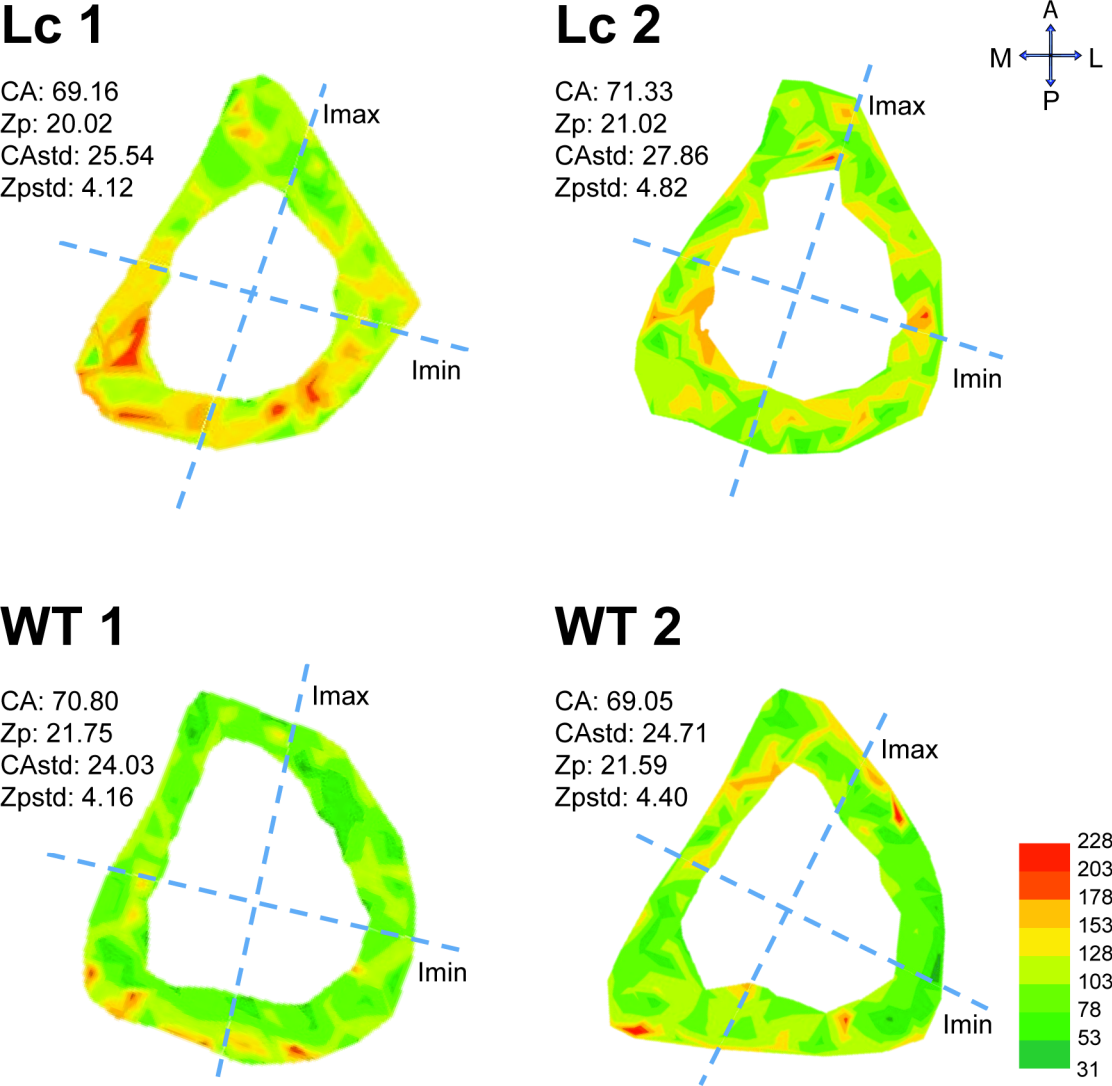
The distribution of N.Ca within a cross section of two Lc and two WT mice from the second data set (the color scale indicates the frequency of the N.Ca that range from the minimum (green) to maximum (red) frequencies; see abbreviation and units in Tables 1 and 2).

The distribution of N.Ca within a cross section of two Lc and two WT mice is shown in Fig 4. Supporting the previous results based on the second data set, the Lc lacunae have more radiating canaliculi compared with the WT lacunae. In addition, the N.Ca within the Lc lacunae is decreased in the I-regions I_max__A and increased in the endosteal H-regions. The N.Ca within the WT lacunae is increased in the I-regions I_max__P and the periosteal H-regions.

### Lacunar volume

Table 2 summarizes also the differences in the lacunar volume (Lac.V) between the Lc and WT samples divided by the I-regions and H-regions. There are no significant differences in the Lac.V between the Lc and WT mice when averaged over all regions or in all studied I-regions (Fig 3B). The Fisher’s LSD post-hoc tests indicate that the Lac.V within both Lc and WT mice is significantly increased in the region I_max__P compared with the other I-regions. According to the H-regions, the Lc mutants exhibit significantly lower (*p* = 0.047) Lac.V in the periosteal region compared with the WT controls (Fig 3E). The Fisher’s LSD post-hoc tests indicate that the midcortical region within both the Lc and WT mice has significantly increased Lac.V compared with the other H-regions.

### Orientation

Table 2 also shows the differences in the lacunar orientation between the Lc and WT samples by the I-regions and H-regions. The values of orientation are significantly lower (*p* = 0.010) in the Lc mutant mice when averaged over all regions. The Lc mutant mice have also significantly lower values of orientation in the regions I_max__A (*p* = 0.010) and I_min__L (*p* = 0.004) compared with the WT controls (Fig 3C). The Fisher’s LSD post-hoc tests indicate that the region I_min__M within the WT controls has significantly lower values of orientation compared with the regions I_max__A and I_min__L. According to the H-regions, the Lc mutants exhibit lower values of orientation in the midcortical region compared with the WT controls (Fig 3F). The Fisher’s LSD post-hoc tests indicate that the midcortical region within both the Lc and WT mice has significantly increased orientation values compared with the other H-regions.

### Lacuna to lacuna distance

**Fig 5:**
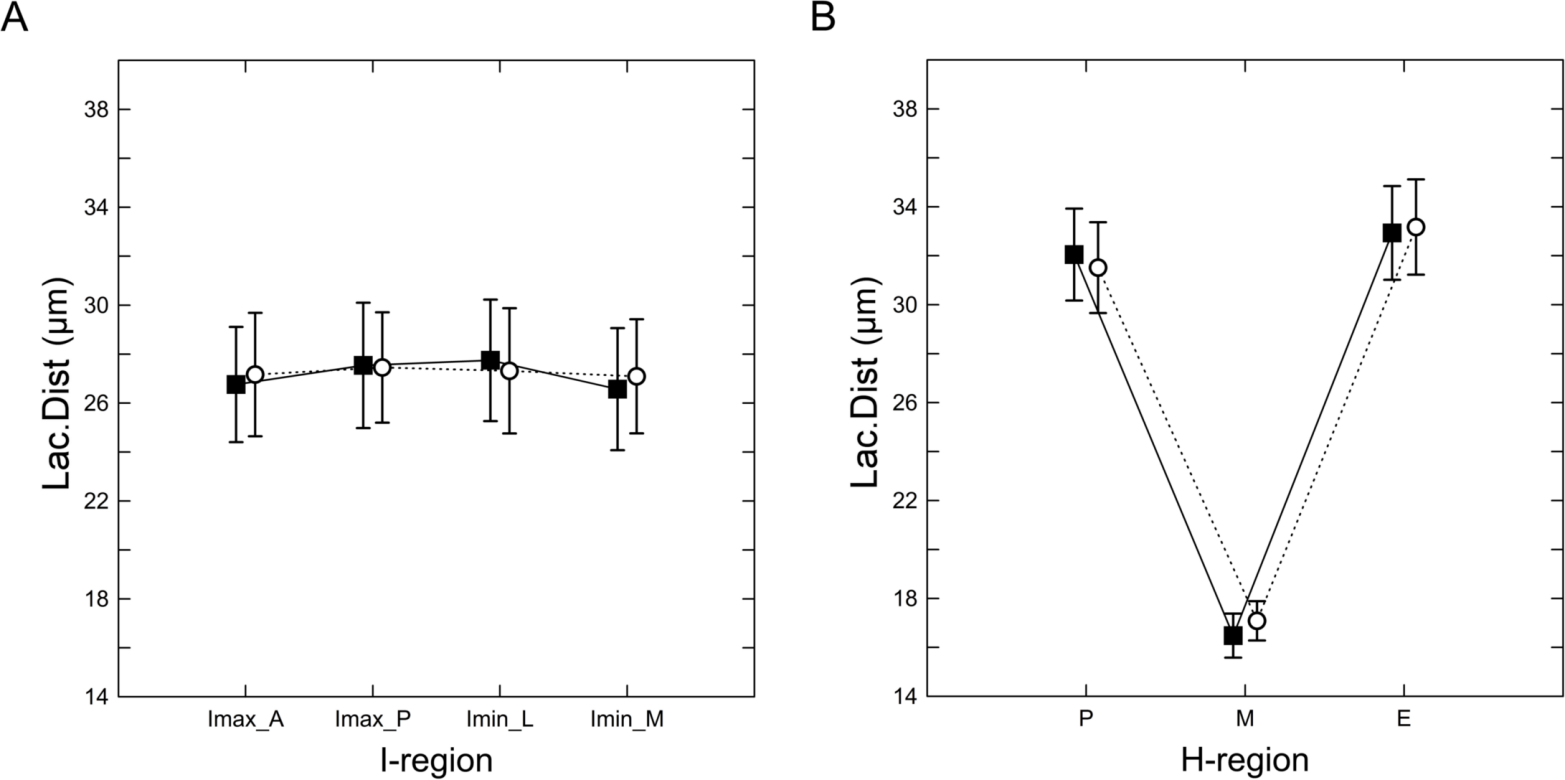
The mean lacuna to lacuna distance by the (A) I-regions and (B) H-regions between the Lc mutants (open circles) and WT controls (closed squares) from the second data set. Vertical bars denote the 0.95 confidence intervals of the means.

Table 3 shows the differences in the lacuna to lacuna distance (Lac.Dist) between the Lc and WT samples by the I-regions and H-regions. There is no significant difference in the Lac.Dist between the Lc and WT mice when averaged over all regions, in all studied I-regions (Fig 5A) and H-regions (Fig 5B). Fisher’s LSD post-hoc tests indicate that the midcortical regions within both the Lc and WT groups of mice have a significantly smaller Lac.Dist compared with the other H-regions.

**Table 3:** Descriptive statistics for the lacuna to lacuna distance (Lac.Dist) between the Lc mutants (n = 5) and WT controls (n = 5) and the pooled mouse sample (letters in superscript indicate the Fisher’s LSD post-hoc significances between the I- and H-regions at *p* < 0.05).

Region	Average lacuna to lacuna distance (μm)			
	Pooled mouse sample	*Lurcher*	Wild type	*p*-value
All regions	27.20	27.26	27.15	0.875
I_max__A	26.96	27.16	26.76	0.757
I_max__P	27.50	27.45	27.54	0.947
I_min__L	27.53	27.32	27.74	0.743
I_min__M	26.83	27.10	26.57	0.689
Periosteal	31.78^f^	31.51^f^	32.05^f^	0.637
Midcortical	16.78^e^^,^^g^	17.08^e^^,^^g^	16.48^e^^,^^g^	0.693
Endosteal	33.06^f^	33.17^f^	32.93^f^	0.833

^e^ periosteal

^f^ midcortical

^g^ endosteal.

LCN properties in the pooled mouse sample

Tables 3 and 4 summarize the differences in the number of canaliculi (N.Ca), lacunar volume (Lac.V), orientation and lacuna to lacuna distance (Lac.Dist) in the pooled mouse sample by the I-regions and H-regions. Fisher’s LSD post-hoc tests indicate that the N.Ca in the pooled mouse sample is significantly increased in the I-regions I_max__P and I_min__M compared with the other I-regions and significantly lower in the midcortical compared with the other H-regions. The Lac.V in the pooled mouse sample is significantly increased in the I-region I_max__P and lower in the I_min__L compared with the other I-regions and significantly increased in the midcortical compared with the other H-regions. The orientation values are significantly increased in the midcortical region compared with the other H-regions. In addition, the Lac.Dist in the pooled mouse sample is significantly lower in the midcortical region compared with the other H-regions.

**Table 4:** Descriptive statistics for the number of canaliculi (N.Ca), lacunar volume (Lac.V) and orientation in the pooled mouse sample (letters in superscript indicate the Fisher’s LSD post-hoc significances between the I- and H-regions at *p* < 0.05).

Region	n lacunae	Average N.Ca	Average Lac.V (μm^3^)	Average orientation (°)
I_max__A	334	104.27^b^^,^^d^	328.38^b^^,^^c^	19.19
I_max__P	299	121.51^a^^,^^c^	374.95^a^^,^^c^^,^^d^	16.76
I_min__L	276	108.67^b^^,^^d^	292.02^a^^,^^b^^,^^d^	19.16
I_min__M	251	117.65^a^^,^^c^	324.36^b^^,^^c^	16.99
Periosteal	383	117.28^f^	302.72^f^	14.11^f^
Midcortical	329	98.31^e^^,^^g^	403.00^e^^,^^g^	27.51^e^^,^^g^
Endosteal	448	119.24^f^	301.95^f^	14.55^f^

^a^ Imax_A

^b^ Imax_P

^c^ Imin_L

^d^ Imin_M

^e^ periosteal

^f^ midcortical

^g^ endosteal.

### Bivariate distribution of N.Ca, Lac.V against C.Dist and N.Ca against Lac.V

**Fig 6:**
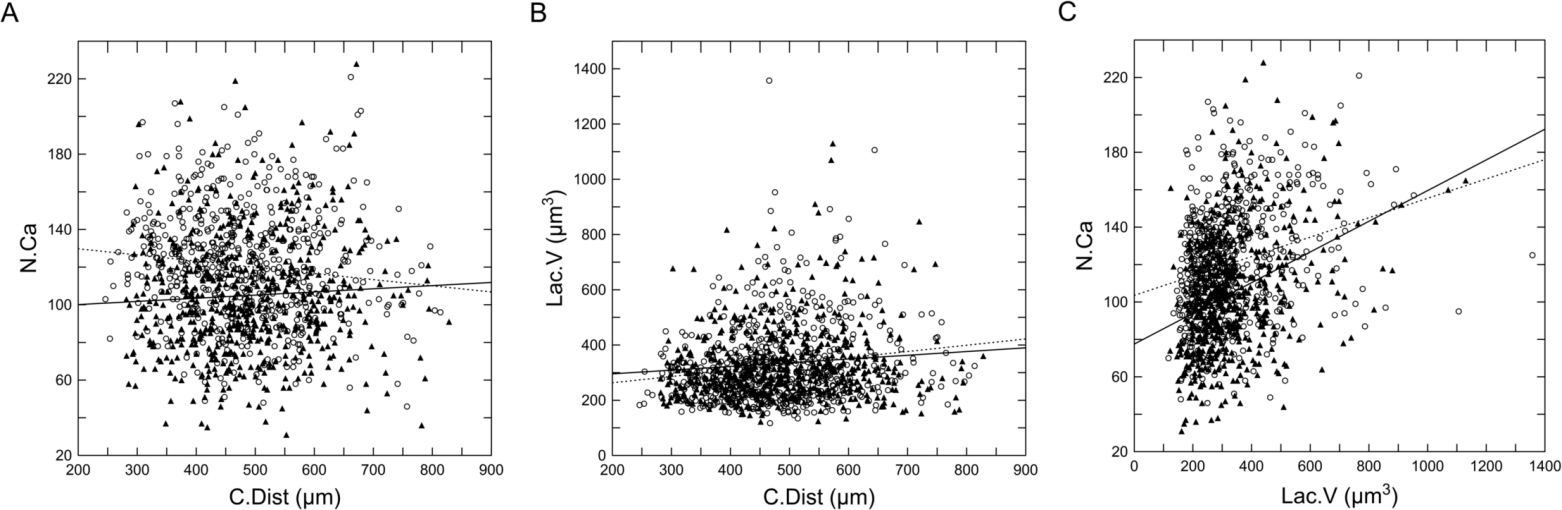
The mean lacuna to lacuna distance by the (A) I-regions and (B) H-regions between the Lc mutants (open circles) and WT controls (closed squares) from the second data set. Vertical bars denote the 0.95 confidence intervals of the means.

Fig 6A shows the bivariate distribution of the N.Ca against the C.Dist, Fig 6B shows the bivariate distribution of the Lac.V against the C.Dist and Fig 6C shows the bivariate distribution of the N.Ca against the Lac.V in the Lc mutants and WT controls from the second data set. The N.Ca is significantly correlated with the distance of the lacuna from the centroid in both the Lc and WT mice; however, the slopes of the regression line indicate different allometric patterns for the Lc and WT mice (Fig 6A). The N.Ca in the Lc mutants is decreased with the distance from the centroid, whereas the N.Ca in the WT controls is increased. The distance from the centroid has no effect on the Lac.V in both the Lc and WT mice (Fig 6B). The bivariate distribution of N.Ca against Lac.V is presented in Fig 6C. The results indicate a positive correlation between the variables in both analyzed groups of mice.

## Discussion

To the best of our knowledge, this study is the first study to assess the effects of a neurodegenerative motor disorder on cortical bone tissue at both the structural (CSG) and microstructural (LCN) levels in *Lurcher* mice. Lc mice have exhibited a significant decrease in motor performance in previous studies [35–42, 63], as well as in our studied sample. Therefore, we investigated the effects of altered mechanical loadings on the structural and microstructural properties of the tibial transverse mid-diaphyseal cross section between Lc mutant and WT control mice. At the structural level, we identified inconsistent differences in the CSG properties between the Lc mutants and WT controls in the case of the appropriate size standardized CSG properties. At the microstructural level, the most significant difference in the LCN properties between the Lc and WT mice was identified in N.Ca, whereas there were only limited differences in the other LCN properties. However, if the LCN properties were studied in the pooled Lc and WT samples, significant differences in the bone microstructure with respect to the second moment of area axes (I-regions) and histological regions (H-regions) were identified.

The CSG properties are non-significant between the Lc mutants and WT controls, with the exception of the smaller CAstd in the 9M WT mice. It is unlikely that these inconsistent differences in the size standardized CSG properties between the Lc mutants and WT controls are caused by the attainment of the final adult structural CSG properties before the progressive motor degenerative disorder reaches the final stage (approximately 30 days of age) [34]. It has been demonstrated that body size and CSG properties increase even through adulthood [64–66]. The increase in the body size and cortical parameters is also supported, in part, by the absolute increase of the BM, CA, and Zp between the 4M and 9M mice in our sample (although only the Lc CA and Zp are significant between the 4M and 9M).

On the other hand, the non-significant size standardized CSG properties between the Lc mutants and WT controls may indicate that the motor differences in Lc mutants produce different loadings only within the maximum and minimum effective values proposed in the mechanostat by H.M. Frost [67]. The structural alterations in the CSG properties in mice have mainly been demonstrated in experimental setting on the level of the mechanical loading, which significantly exceeded the normal physiological loading experienced by animals during normal living conditions. For example, Souza et al. [68] demonstrated that only the loads greater than 10N (i.e., approximately 6 × higher loadings than produced by normal walking) caused new bone formation in the tibial mid-diaphysis in their mouse sample. Similarly, the minimum effective loading was observed on the non-physiological unloading of long bones induced by tail-suspension [69] or sciatic neurectomy [70]. These loading magnitudes are far away from the expected habitual loading of the Lc mouse models. On the other hand, the structural alterations in the CSG properties have been also demonstrated using physiological loading models, for example exercise/running models [71]. The study by Styner et al. [71] showed increased bone quantity in exercise groups of mice that had been provided voluntary access to running wheels. However; in order to study also the bone microstructural level of the exercise/running models further research is required.

At the microstructural level, the most significant difference between Lc and WT mice is found in N.Ca whereas the other LCN properties show only limited differences. In general, the Lc mutant mice had more N.Ca in all studied I- and H-regions of the cross sections compared with the WT controls. Moreover, the N.Ca was negatively correlated with the distance from the centroid in the Lc mutants and positively correlated in the WT controls. As the osteocyte processes within the canaliculi may play a role in the mechanosensing [72, 73], this variation in N.Ca may be the adaptations for differential mechanical requirements in the habitual loading of the mutant Lc and healthy WT mice. The key factors regulating osteocyte network formation are not well understood; however, we suggest that the cerebellar ataxia and related motor changes including dynamic tremor in Lc mice may in general increase osteocytes activity, which can be connected with the increased number of canaliculi observed in this study. Considering that osteocytes are connected and communicate with each other, with surrounding lining cells on the bone surface and cells within the bone marrow [47, 50, 74], it is suggested that higher mechanical requirements of the bone tissue may increase number of processes ensuring rapid transmission of signals [47]. In our study, the average N.Ca that radiates from a particular lacuna was 112 (average number of canaliculi that ranged from 92 located midcorticaly to 115 located periostealy). Our results are somewhat different from other reported values. For example, Sugawara et al. [49] identified a higher average number of processes (N.Ca = 55) in healthy and a lower value (N.Ca = 45) in an immobilized group of adult mice when the sample was obtained from the periosteal region of the femora. Hirose et al. [18] measured the N.Ca between 17 and 18 in the periosteal region and between 16 and 20 in the endosteal region of the tibia in developing mice. However, it is likely that these differences between our results and the observed N.Ca in other analyses are because of a localization effect of the studied lacunae within the I- and H-regions. To the best of our knowledge, this study is the first time that the number of canaliculi was measured in lacunae randomly selected within the entire mouse cross section with different histological localizations and biomechanical properties. Moreover, it is unclear whether the observed differences are also an effect of differences in loading between samples or that the properties of the lacunae are not homogenous in the cross section and the results reflect differences in variations within the H- and I-regions.

In addition to the limited significant differences in the LCN properties between the Lc mutants and WT controls, we identified significant differences in the bone microstructure level within the studied I- and H-regions in the pooled mouse sample. In the I-regions, the I_max__P exhibited lacunae with more radiating canaliculi and a greater volume and the I_min__L exhibited a smaller volume. Within the H-regions, the midcortical region exhibited LCN that contained larger lacunae, which were located closer to each other with a lower number of canaliculi. Conversely, the lamellar bone located beneath the periosteal and endosteal surfaces had smaller lacunae, which were situated further from each other, and had more canaliculi that radiated from single lacunae. The non-homogenous LCN distribution in mice has also been noted in other studies [18, 52]. Thus, our results highlight the importance of precise sample selection within transversal cross sections used for lacunar-canalicular analysis in future mouse studies and careful consideration of the obtained observations with respect to the differences in the H- and I-regions.

Besides the altered motor performance, the Lc mutants also show hyper-reactivity of the hypothalamic-pituitary-adrenal axis, while basal levels of both adrenocorticotropic hormone and corticosterone are similar in *Lurcher* mutants and control mice [75]. Thus, exposure to an anxiogenic situation increases corticosteron levels more in the mutants than in controls [75]. High corticosterone levels inhibit bone formation [76]. Since the mice used in our study were not deliberately exposed to the anxiogenic situations, we expect a low effect of the stress-provoked high corticosterone levels.

Unlike the known altered motor performance in the Lc mutant mice, the bone mechanical loading differences in Lc mice have not been directly measured yet. Further research of mechanical loading distribution within the cross-section would help to better understand the Lc bone functioning and to determine the relation between the currently observed regional microstructural variation and the local mechanical loading environment.

In conclusion, at the structural level, we identified inconsistent differences in the CSG properties between Lc mutants and WT controls. At the microstructure level, we identified significant differences within the studied I- and H-regions in the pooled Lc and WT sample, and the strongest difference between the Lc and WT mice was identified in the number of canaliculi. This research highlights the regional spatial LCN heterogeneities in the mouse tibial mid-diaphyseal cross section. Our findings indicate that in future studies, the mouse bone samples for LCN analysis should be obtained, and the results should be interpreted with regards to these regional differences.

## Supporting Information

S1 File The raw data from the CSG analysis. (XLSX)

S2 File The raw data from the LCN analysis. (XLSX)
